# Right-Sided Urinary Extravasation Caused by a Ureteral Stone and Associated With Peritonitis in an Older Woman

**DOI:** 10.7759/cureus.34174

**Published:** 2023-01-24

**Authors:** Yumi Naito, Soshi Takagi, Keita Inoue, Chiaki Sano, Ryuichi Ohta

**Affiliations:** 1 Communiy Care, Unnan City Hospital, Unnan, JPN; 2 Family Medicine, Shimane University Medical School, Izumo, JPN; 3 Urology, Unnan City Hospital, Unnan, JPN; 4 Community Medicine Management, Shimane University Faculty of Medicine, Izumo, JPN; 5 Communiy Care, Unnan City Hospital, Unnan, Shimane, JPN

**Keywords:** general medicine, gerota fascia, peritonitis, abdominal pain, urinary extravasation

## Abstract

Right upper quadrant pain can originate from the liver, cholecystic duct, gallbladder, pancreas, or surrounding organs. Peritonitis in the right upper quadrant of the abdomen can be caused by lesions in these organs as well as the adjacent organs, such as the kidney and colon. The kidneys are surrounded by Gerota’s fascia and fat; therefore, mild local inflammation may not cause peritonitis. Herein, we report the case of a 72-year-old woman with right-sided abdominal pain who was diagnosed with urinary extravasation due to a ureteral stone. Urinary extravasations can present with peritonitis. For effective diagnosis, prompt physical examination and abdominal ultrasound are essential, with the extent of extravasation being key to effective management. Therefore, general physicians should consider urinary extravasation, which is typically caused by kidney and urinary stones, in patients with right upper quadrant pain.

## Introduction

Abdominal pain can originate from various organs, so localized pain and tenderness can help confirm the involved organs. Right upper quadrant pain can be caused by the liver, cholecystic duct, gallbladder, pancreas, and surrounding organs [1]. Common diseases in this quadrant include acute hepatitis, cholangitis, cholecystitis, and pancreatitis [2,3], which can cause peritonitis locally through the direct spread of inflammation from the organs to the peritoneum [4]. Peritonitis can manifest as persistent pain with gourding, rebound tenderness, and percussion pain upon physical examination. These findings are important for the diagnosis of patients with right-quadrant abdominal pain.

Peritonitis in the right upper quadrant of the abdomen can also be caused by adjacent organs such as the kidney and colon [5]. Gerota’s fascia and fat surround the kidneys, and mild local inflammation may not cause peritonitis [6]. However, a severe inflammatory condition of the kidneys may cause upper quadrant abdominal peritonitis [7]. We encountered an elderly patient with right-sided abdominal pain who was diagnosed with urinary extravasation due to a right urinary stone. This disease may have been benign; however, in our case, positive signs of Murphy’s sign and severe peritonitis were observed at presentation. By reporting this case, we aim to show the importance of considering urinary extravasation in the differential diagnosis of right upper quadrant peritonitis and discuss its pathophysiology.

## Case presentation

A 72-year-old woman was admitted to a rural Japanese community hospital with the chief complaints of right lower abdominal pain and appetite loss. One day before the admission, she experienced acute and continual right lower quadrant abdominal pain at night. The pain was insidious at the onset and progressed slowly. The pain was exacerbated with deep breathing, coughing, and postural changes. The patient had no history of abdominal trauma. Her medical history included hypertension, reflux esophagitis, and peripheral vertigo, for which she was prescribed irbesartan, azelnidipine, vonoprazan, and merislon, once a day.

At presentation, her vital signs were as follows: blood pressure, 132/74 mmHg; heart rate, 80 beats/min; body temperature, 37.0 °C; respiratory rate, 18 breaths/min; and SpO2, 96% on ambient air. She was conscious and well-oriented. On physical examination, her abdomen was flat and soft, with tenderness in the right upper quadrant and flank and pain on percussion. Ultrasonography revealed dilation of the right renal pelvis and a hypoechoic region around the kidneys. The white blood cell count was mildly elevated, renal parameters worsened (0.72 to 1.15 mg/dL) compared to her blood test results three months ago at her primary care clinic, and urinalysis showed microhematuria (Table 1).

**Table 1:** Initial laboratory data of the patient

Parameters	Value	Reference
White blood cell count	9.30	3.5–9.8 × 10^3^/μL
Red blood cell count	4.40	4.10–5.30 × 10^6^/μL
Hemoglobin	12.6	13.5–17.6 g/dL
Hematocrit	37.4	36–48%
Mean corpuscular volume	92.6	82–101 fl
Platelet count	12.2	13.0–36.9 × 10^4^/μL
Total protein	6.9	6.6–8.1 g/dL
Serum albumin	4.0	3.9–4.9 g/dL
Total bilirubin	1.0	0.2–1.2 mg/dL
Serum aspartate aminotransferase	146	8–38 IU/L
Serum alanine aminotransferase	137	4–44 IU/L
Serum γ-glutamyl transpeptidase	379	16–73 IU/L
Serum lactate dehydrogenase	248	106–211 U/L
Blood urea nitrogen	20.4	8.0–20.0 mg/dL
Serum creatinine	1.15	0.40–1.10 mg/dL
Estimated glomerular filtration rate	36	>60.0 mL/min/L
Serum sodium	135	135–147 mEq/L
Serum potassium	3.9	3.3–4.8 mEq/L
Serum chloride	101	98–108 mEq/L
Urine analysis		
White blood cells	Negative	Negative
Nitrite	Negative	Negative
Protein	Negative	Negative
Glucose	Negative	Negative
Urobilinogen	Negative	Negative
Bilirubin	Negative	Negative
Ketone bodies	Negative	Negative
Blood	Negative	Negative
pH	7	
Specific gravity	1.02	

**Figure 1:**
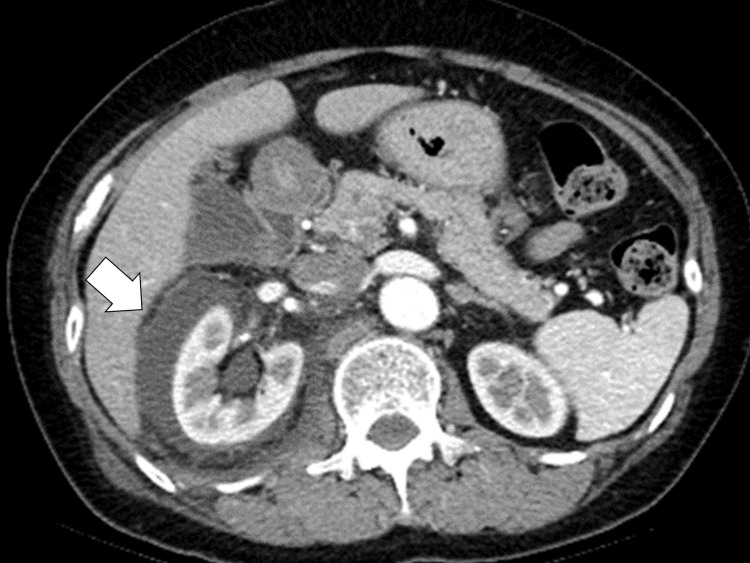
Contrast-enhanced abdominal computed tomography showing leakage of contrast medium during the excretory phase around the right kidney (white arrow)

Contrast-enhanced abdominal computed tomography (CT) showed leakage of contrast around the right kidney during the excretory phase (Figure 1).

A 3 mm stone was identified in the right upper ureter. Based on these findings, the patient was diagnosed with right-sided urinary extravasation caused by a ureteral stone. The 3 mm ureteral stones were treated with adequate intravenous hydration and diclofenac. The following day, the pain improved, and abdominal ultrasonography showed resolution of the hypoechoic region around the kidneys. Blood tests performed on the fourth day of hospitalization showed improved renal function (1.15 to 0.82 mg/dL). A simple abdominal CT scan on the same day revealed that the ureteral stone and urine overflow around the kidneys had resolved. The patient was discharged from the hospital on the seventh day.

## Discussion

This report describes a case of right urinary extravasation due to a ureteral stone. Physical examination revealed tenderness in the right upper quadrant, suspecting of peritonitis. Various diseases can cause right upper abdominal pain and peritoneal irritation. Although gastrointestinal disorders such as cholangitis and cholecystitis were considered when the patient complained of right upper quadrant pain with peritoneal irritation, it was important to differentiate between these diseases.

The relationship between urinary tract tissues regarding internal pressure can explain the pathophysiology of urinary extravasation. In our case, when renal pelvic pressure rises due to stone impaction, it causes microscopic fissures in the anatomically weakest part of the renal calyx [8,9]. As a result, urine overflows outside the renal pelvis. This is considered a safety mechanism that protects the kidneys from excessive pressure [6]. Normal renal pelvic pressure is <10 mm Hg and can absorb up to 30-45 mmHg. Thus, pressures >30-45 mmHg cause extrarenal overflow [10]. Although extrarenal overflow may occur at 20 mmHg, it may not occur at 100 mmHg [8,9]. This indicates that the speed of increase in renal pelvic pressure is more important than absolute renal pelvic pressure. In our case, acute onset impaction of the right ureter might increase the pressure of the renal pelvis, causing fissures in the urinary tract and extravasation of urine.

The average age at which urinary extravasation occurs is 53 years, with men and the left side more likely to be affected than women and the right side [8,9]. Ureteral stones are the most common cause, followed by urogenital and extra-urinary tumors [10]. Our patient was a woman who developed right urinary extravasation due to a ureteral stone and did not show hematuria as well, so the presentation is atypical. Approximately 48% of patients with ureterolithiasis develop spontaneous extrapelvic extravasation; thus, this may be a relatively common disease that is not well known [10].

The symptoms of urinary extravasation vary and can complicate the diagnostic process. In a review of 21 studies, the most common symptom in 66 patients was flank pain (56%), followed by back pain (15%), and lower abdominal pain (13%) [11]. Although this patient had peritonitis similar to cholangitis, symptoms of peritoneal irritation were observed in 4% of cases [11], while right lower abdominal pain and peritoneal irritation were relatively rare. Stimulation of the sympathetic nervous system in the renal fascia due to rapid enlargement of the kidney and ureteral obstruction reportedly causes flank, back, and lower abdominal pain [11]. Peritoneal irritation symptoms may also be caused by tension in the perirenal Gerota’s fascia due to urine overflow or radiation of the pain to the abdomen [12]. Gastrointestinal diseases and extrapelvic urinary overflow can cause peritoneal irritation. In addition to confirming the presence of ascites on ultrasonography, it is also important to pay attention to the presence of a hypoechoic area around the kidneys when a patient complains of vague abdominal pain [13].

## Conclusions

Urinary extravasation has various symptoms and can sometimes present with peritonitis. For effective diagnosis, prompt physical examination and abdominal ultrasound are essential. The detection of the extravasation is the key to an effective diagnosis; therefore, general physicians should consider this disease, which is typically caused by kidney and urinary stones, in patients presenting with right upper quadrant pain.
